# Intraoperative hypotension during critical phases of liver transplantation and its impact on acute kidney injury: a retrospective cohort study

**DOI:** 10.1016/j.bjane.2024.844566

**Published:** 2024-10-16

**Authors:** Matthanja Bieze, Amir Zabida, Eduarda Schutz Martinelli, Rebecca Caragata, Stella Wang, Jo Carroll, Markus Selzner, Stuart A McCluskey

**Affiliations:** a Toronto General Hospital, Department of Anesthesia and Pain Management, Toronto, Ontario, Canada; b University of Toronto, Temerty Faculty of Medicine, Department of Anesthesiology and Pain Medicine, Toronto, Ontario, Canada; c Austin Health, Department of Anesthesia, Melbourne, Australia; d University of Melbourne, School of Medicine, Department of Critical Care, Melbourne, Australia; e University Health Network, Department of Biostatistics, Toronto, Ontario, Canada; f Temerty Faculty of Medicine, Toronto General Hospital, Department of Surgery, and the Multi-Organ Transplant Program, Toronto, Ontario, Canada

**Keywords:** Acute kidney injury, Blood pressure, Hypotension, Liver transplantation, Postoperative complications, Reperfusion

## Abstract

**Introduction:**

Acute Kidney Injury (AKI) following Liver Transplantation (LT) is associated with prolonged ICU and hospital stay, increased risk of chronic renal disease, and decreased graft survival. Intraoperative hypotension is a modifiable risk factor associated with postoperative AKI. We aimed to determine in which phase of LT hypotension has the strongest association with AKI: the anhepatic or neohepatic phase.

**Methods:**

This retrospective cohort study included adult patients undergoing LT between January 2010 and June 2022. Exclusion criteria were re-do or combined transplantations, preoperative dialysis, and early graft failure or death. Primary outcome was AKI as defined by KDIGO. Hypotension was Mean Arterial Pressure (MAP) below predefined thresholds in minutes. Risk adjusted logistic regression analysis considered hypotension in 3 periods: the total procedure, anhepatic phase, and neohepatic phase.

**Results:**

Our cohort included 1153 patients. The median MELD-NA score was 19 (IQR 11–28), and 412 (35.9%) were living-related donations. AKI occurred in 544 patients (47.2%). The unadjusted model showed an association with AKI for MAP < 60 mmHg (OR = 1.011 [1.0, 1.022], *p* = 0.047) and MAP < 55 mmHg (OR = 1.023 [1.002, 1.047], *p* = 0.040) in the anhepatic phase, and for MAP < 60 mmHg (OR = 1.032 [1.01, 1.056], *p* = 0.006) in the neohepatic phase. The adjusted model did not reach significance in the subgroups but did in the total procedure: MAP < 60 mmHg (OR = 1.005 [1.002, 1.008], *p* < 0.001) and MAP < 55 mmHg (OR = 1.008 [1.003–1.013], *p* = 0.004).

**Conclusion:**

Intraoperative hypotension is independently associated with AKI following LT. This association is seen during the anhepatic phase. Maintaining MAP above 60 mmHg may improve kidney function after LT.

## Introduction

Acute Kidney Injury (AKI) after Liver Transplantation (LT) occurs in 41%–50% of cases, of which 7%–15% need renal replacement therapy.^1^, ^2^, ^3^, ^4^ Patients with AKI have a worse outcome after LT with a prolonged ICU and hospital stay, increased chronic renal disease, and worse graft survival.^2^^,^^5^ Most factors associated with AKI are not modifiable at time of the transplant, including recipient baseline renal function, obesity, diabetes mellitus, MELD score, donor age, graft steatosis, and donation after circulatory death.^6^, ^7^, ^8^ However, intraoperative hypotension is a potential modifiable risk factor when the duration and target of blood pressure at which end organ damage occurs can be identified. We recently found that a Mean Arterial Pressure (MAP) of < 55 mmHg for a duration of > 20 minutes is associated with an increased risk of postoperative AKI.^9^ To narrow down the most critical phase of hypotension during LT, we must consider the three phases of LT: dissection phase, anhepatic phase, and neohepatic phase, also referred to as reperfusion phase. Of them, the anhepatic and the neohepatic phases are thought to have the greatest impact on kidney function. During the anhepatic phase kidney function may be compromised by the increase in venous pressure against the caval cross-clamp and the decreased arterial pressure leading to compromised renal perfusion pressures.^10^ During the neohepatic phase the hepatic reperfusion injury is thought to compromise renal function with the release of catecholamines, ischemic metabolites, and potassium from the donor liver, combined with suboptimal graft function and Postreperfusion Syndrome (PRS).^11^

With the current study, our objective was to confirm the association between hypotension and AKI and to determine whether the critical hypotension occurred in the anhepatic or neohepatic phase of liver transplantation, to give the clinician modifiable objectives to improve postoperative kidney function.

## Methods

This is a retrospective single center cohort study in patients undergoing liver transplantation in a tertiary academic hospital in Canada between January 1, 2010 and June 30, 2022. Exclusion criteria were combined kidney-liver transplant or multi-visceral transplant, preoperative dialysis, early graft failure with re-do or mortality, and absence of digital hemodynamic data or perioperative serum Creatinin (sCR) levels. The study was conducted in accordance with standard ethical considerations and the University Health Network Research Ethics Board approved the study protocol (CAPCR 20-5974) and a waiver for informed consent was granted. The records of adult patients who underwent liver transplantation were screened for eligibility and data were extracted from the electronic patient record system and clinical databases which are part of the standard documentation in our institution. All patients underwent invasive blood pressure monitoring during LT and these data were recorded from the moment the patient was connected to the monitor in the operating room until the patient transferred out of the operating room. The MAP values were extracted and cleaned from outliers consistent with artifacts (< 0 or > 220 mmHg). The duration in minutes the individual patient remained under different MAP intervals (MAP of 70, 65, 60, 55, 50, 45 and 40 mmHg) was calculated and the intervals used were similar to those used in previous non-cardiac surgery studies.^12^, ^13^, ^14^

The primary outcome was the occurrence of AKI during the first seven days after liver transplantation. AKI was defined according to the Kidney Disease Improving Global Outcomes (KDIGO) criteria for serum creatinine: an absolute increase in sCr ≥ 26.5 mcmoL.L^-1^ (0.3 mg.dL^-1^) above baseline value or a relative increase ≥ 1.5 times baseline value. Baseline sCR was considered the value immediately prior to transplantation. Urine output criteria were not considered. Secondary outcome was the exposure to intraoperative hypotension, defined as a time duration (in minutes) below MAPs of 75, 70, 65, 60, 55, 50, 45, and 40 mmHg. The anhepatic phase was defined as the time from caval clamping until unclamping. The neohepatic phase started at the moment the caval clamp was released until 30 minutes post reperfusion of the graft. Covariates were baseline patient factors (age, sex, height and weight), comorbidities (including Etiology of end Stage Liver Disease (ESLD), MELD score, and complications of liver disease), perioperative laboratory values, donor graft details (Neurological Determination of Death (NDD), Donation after Cardiac Death (DCD), living donor), the use of Normothermic Machine Perfusion (NMP)(OrganOx),^15^ and intraoperative characteristics (surgical anastomosis type [piggyback or caval interposition], cross-clamp type [partial or complete], cold and warm ischemic times, and transfusion details).

Due to the exploratory character of the study, the sample size was not formally calculated. A post-hoc power calculation shows that given our sample size of 1153 and 544 AKI events, we were able to estimate the proportion of AKI of 47% with 3% absolute precision and 95% confidence. Statistical analyses were performed using the R software (Version 4.2.1). Univariable logistic models were used to test for association between MAP exposures and AKI as well as the covariates and AKI. Multivariable logistic regression was performed, with adjustment for covariates found to have a *p*-value < 0.05 in the univariable analysis. The adjusted Odds (ORs) Ratios were reported with 95% Confidence Intervals and p-values.

To assess the robustness of the results, we performed sensitivity analyses in which we repeated the multivariable regression with AKI in patients with a baseline eGFR > 30 mL.min^-1^ and separately in the group with baseline eGFR > 60 mL.min^-1^. eGFR was estimated using the Cockcroft-Gault equation which was known for all patients in the study.

## Results

### Patient and procedure characteristics

**Figure 1:**
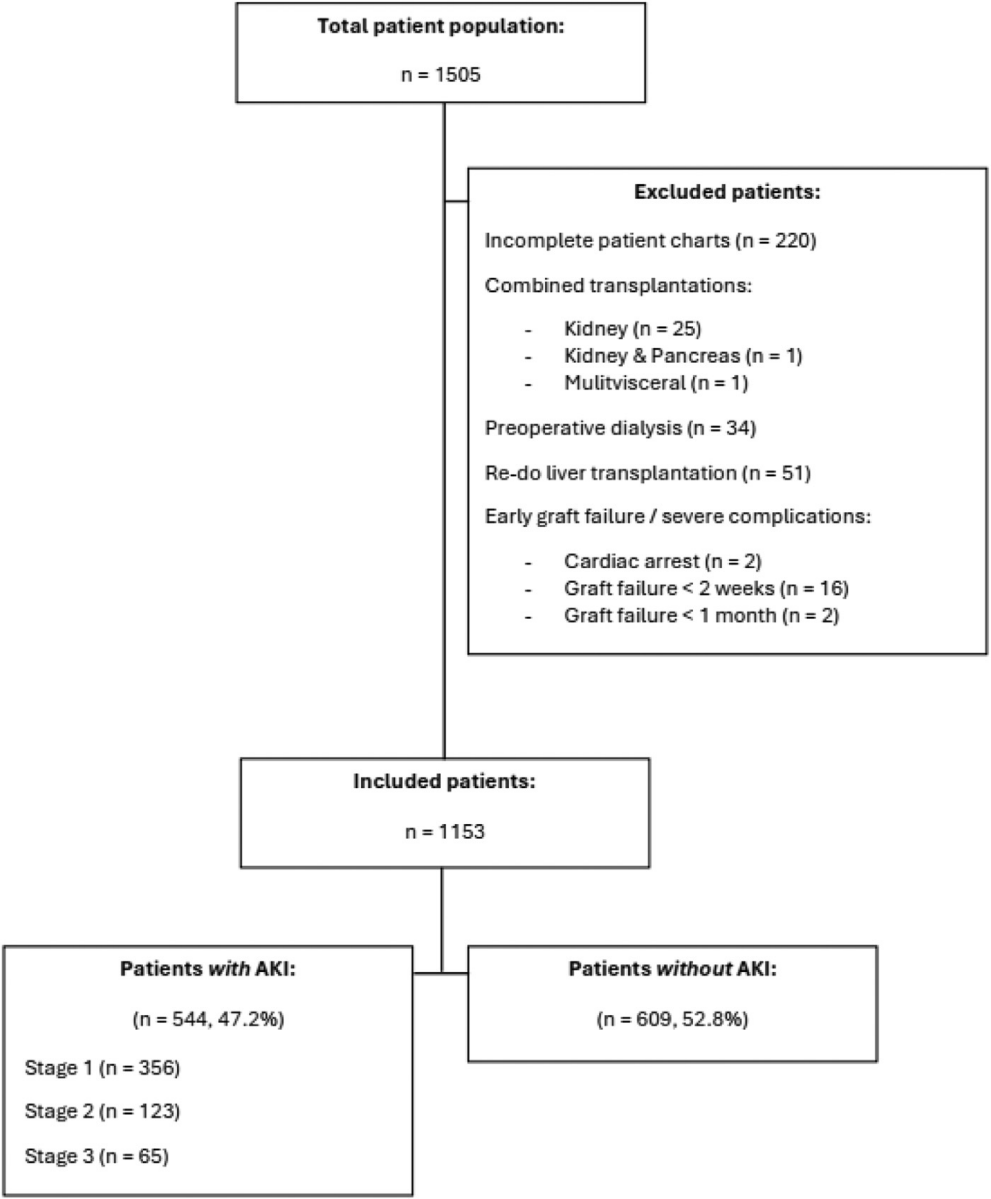
Flowchart patient population. Showing the in- and excluded patients, and the total occurrence of the primary endpoint AKI. In case of multiple transplantations, the first transplantation was included when it was performed within the timeline and criteria of the study.

There were 1505 patients in the total population of which 220 charts were incomplete (missing hemodynamic data) and excluded, 27 patients underwent a combined transplantation, 34 had preoperative dialysis, 51 had a re-do liver transplantation of which only the first transplantation was included when eligible, and 20 patients suffered early graft failure with re-do or mortality and were excluded. In the end, 1153 patients were included in the final patient population (Fig. 1). The etiology of liver disease was metabolic dysfunction associated steatotic liver disease (MASLD) in 242 (21%), hepatitis C in 234 (20.3%), and alcoholic liver cirrhosis in 226 (19.6%) patients. The remaining etiology included primary sclerosing cholangitis (11.1%), hepatitis B (9.7%), primary biliary cirrhosis (6.2%), fulminant liver failure (3.9%), cryptogenic (2.7%), thrombotic (1.2%), and a variety of rare conditions (4.3%). In 473 patients (41%) ESLD was complicated by Hepatocellular Carcinoma (HCC). Median age was 58.6 years [IQR 49.9–64.5], 399 patients were female (34.6%), median BMI was 26.8 [IQR 23.4–30.5], median MELD-NA score was 19 [IQR 11–28]. In 412 (35.9%) patients the donation was living-related. Most grafts were cold static preserved (n = 1121; 98.6%), while 16 (1.4%) patients received a graft after normothermic machine perfusion (OrganOx). Diabetes was present in 258 patients (22.4%) (Table 1).

**Table 1:** Patient characteristics.

Patient characteristics	Total (n = 1153)
**General**	
Age, in median years [IQR]	58.6 [49.9‒64.5]
Gender, female (%)	399 (34.6)
BMI, median [IQR]	26.8 [23.4‒30.5]
Diabetes Mellitus (%)	258 (22.4)
MELD Na, median [IQR]	19 [11‒28]
**Etiology ESLD**	
Acute/Fulminant Hepatic Failure (%)	45 (3.9)
Alcoholic Cirrhosis (%)	226 (19.6)
Cryptogenis Cirrhosis (%)	31 (2.7)
Hepatitis B (%)	112 (9.7)
Hepatitis C (%)	234 (20.3)
Metabolic associated steatotic liver disease (%)	242 (21.0)
Primary Biliary Cirrhosis (%)	71 (6.2)
Primary Sclerosing Cholangitis (%)	128 (11.1)
Thrombotic, including Budd-Chiari (%)	14 (1.2)
Other (including autoimmune hepatitis, Alpha-1 antitrypsin deficiency, Hemochromatosis, Wilson's disease) (%)	50 (4.3)
Presence of Hepatocellular Carcinoma (%)	473 (41.0)
**Laboratory results, preoperative**	
Hemoglobin, median in g.L^-1^ [IQR]	98 [89‒108]
INR, median [IQR]	1.8 [1.6‒2.1]
Sodium, median in mmoL.L^-1^ [IQR]	142 [139‒145]
Creatinine, median in µmoL.L^-1^ [IQR]	92 [74‒125]
Bilirubin, median in µmoL.L^-1^ [IQR]	74 [43‒122]
Albumin, median in g.L^-1^ [IQR]	27 [22‒32]

Patient characteristics. INR, International Normalized Ratio; IQR, Inter Quartile Range; OR, Odd Ratio; CI, Confidence Interval; BMI, Body Mass Index.

Perioperative characteristics included the use of classic caval interposition in 550 (73.7%) and full caval cross-clamp in 699 (79.3%) cases. Cold Ischemic Time (CIT) was 5.7 hours [IQR 2.4–7.9], warm ischemic time was 0.8 hours [IQR 0.6–1.0]. Median blood loss was 2.3 L (IQR 1.4–4.0), median RBC units transfused was three [IQR 0–6], and 256 patients received transfusion of more than 5 RBC units (24.2%) (Table 2).

**Table 2:** Perioperative characteristics.

Perioperative characteristics	Total (n = 1153)
Donor Type	
Living related donation (%)	412 (35.9)
Donation after neurological death (%)	637 (55.5)
Donation after cardiac death (%)	99 (8.6)
Normothermic machine perfusion (%)	16 (1.4)
Cold static preservation (%)	1121 (98.6)
Anastomosis Type	
Classic	550 (73.7)
“Piggy-back”	196 (26.3)
Cross clamp	
Full clamp	699 (79.3)
Partial clamp	182 (20.7)
Cold ischemia time, in hours, median [IQR]	5.7 [2.37‒7.85]
Warm ischemia time, in hours, median [IQR]	0.8 [0.63‒0.98]
Estimated blood loss, in liters, median [IQR]	2.3 [1.4‒4.0]
Red blood cell transfusion, in units, median [IQR]	3 [0‒6]
Red blood cell transfusion > 5 units (%)	256 (24.2)
Acute Kidney Injury (%)	544 (47.2)
Stage 1 (%)	356 (65.4)
Stage 2 (%)	123 (22.6)
Stage 3 (%)	65 (11.9)

Perioperative characteristics and results.

AKI occurred in 544 patients (47.2%). Stage 1 was present in 356 patients (65.4%), Stage 2 in 123 (22.6%), and Stage 3 in 65 (11.9%). After univariable logistic regression the following variables were associated with AKI: male gender (OR = 1.678; 95% CI 1.312–2.150, *p* < 0.001), BMI (OR = 1.082, 95% CI 1.059–1.106, *p* < 0.001), preoperative creatinine (OR = 1.006; 95% CI 1.002–1.011*, p* = 0.009), preoperative bilirubin (OR = 1.004; 95% CI 1.001–1.007, *p* = 0.012), preoperative INR (OR = 2.150; 95% CI 1.276–3.764, *p* = 0.005), MELD Na (OR = 1.023; 95% CI 1.01–1.036, *p* = 0.001), Etiology of Alcoholic Liver Disease (OR = 1.343; 95% CI 1.004–1.799, *p* = 0.047), NDD (OR = 1.502; 95% CI 1.169–1.932, *p* = 0.001), DCD (OR = 2.032; 95% CI 1.306–3.18, *p* = 0.002), CIT (OR = 1.043; 95% CI 1.013–1.077, *p* = 0.008), Blood loss (OR = 1.074; 95% CI 1.03–1.122, *p* = 0.001, and transfusion of > 5 units of RBC (OR = 1.604; 95% CI 1.186–2.178, *p* = 0.002). Surgical technique, including full or partial caval cross-clamp was not significantly different. The PSC group were less likely to develop AKI (OR = 0.509; 95% CI 0.342–0.746, *p* = 0.001). After multivariable regression analysis the following variables remained associated with AKI: male gender (MAP < 60: OR = 1.901 [95% CI 1.4, 2.591], *p* < 0.001; MAP < 55: OR = 1.901 [95%CI 1.402, 2.588], *p* < 0.001; MAP < 50: OR = 1.897 [95% CI 1.400–2.579]), BMI (MAP < 60: OR = 1.076 (95% CI 1.048, 1.106), *p* < 0.001; MAP < 55: OR = 1.076 [95% CI 1.048, 1.106], *p* < 0.001; MAP < 50: OR = 1.075 [95% CI 1.047, 1.104], *p* < 0.001), and MELDNA (MAP < 60: OR = 1.025 [95% CI 1.008, 1.042], *p* = 0.003; MAP < 55: OR = 1.026 [95% CI 1.009, 1.043], *p* = 0.002; MAP < 50: OR = 1.026 [95% CI 1.010, 1.043], *p* = 0.002).

### AKI and MAP thresholds

**Figure 2:**
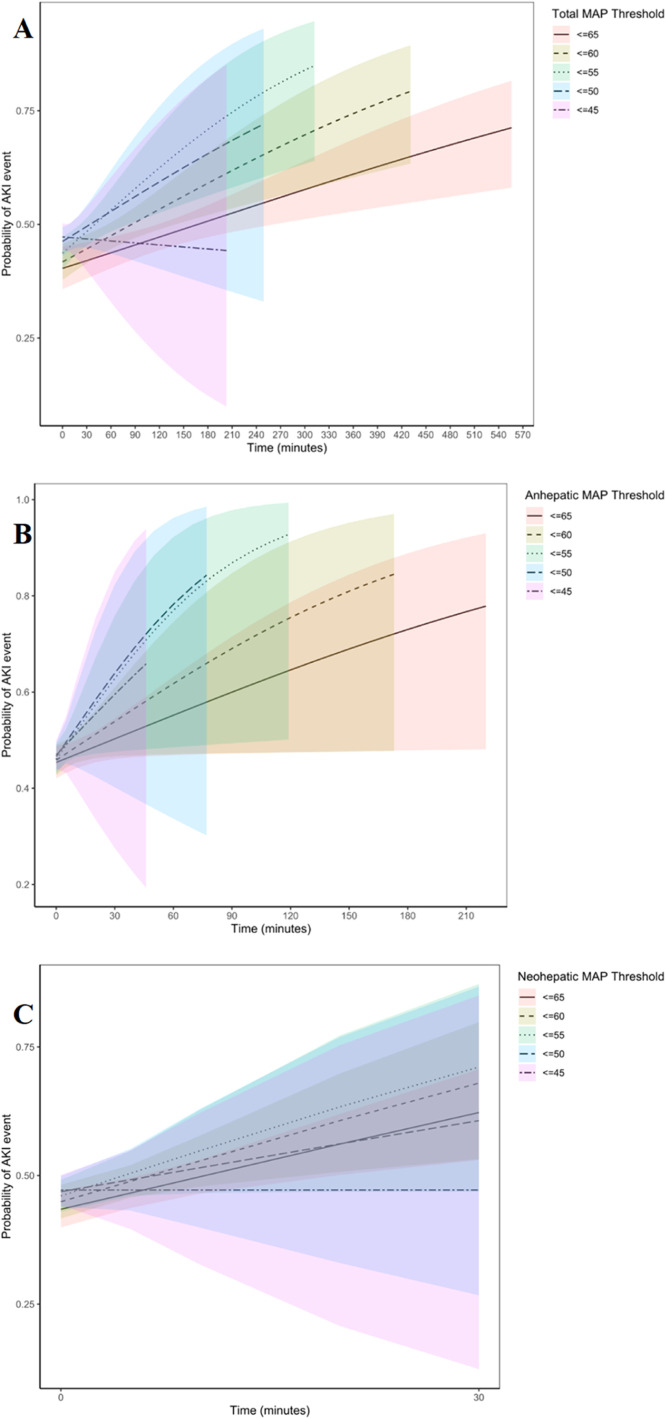
(A) Probability of AKI with different MAP thresholds during the Total Procedure. (B) Probability of AKI with different MAP thresholds during the Anhepatic Phase. (C) Probability of AKI with different MAP thresholds during the Neohepatic Phase. Presenting the predicted probability of an AKI event over hypotension time duration in the total procedure (Fig. 2A), the anhepatic phase (Fig. 2B) and the neohepatic phase (Fig. 2C), modeled using univariable logistic regressions across different MAP thresholds. For each threshold (≤ 65, ≤ 60, ≤ 55, ≤ 50, and ≤ 45 mmHg), the probability of AKI was calculated, and the results are represented by distinct lines and shaded confidence intervals.

When looking at the predicted probability of an AKI event over different MAP thresholds and time duration, the results from the univariable logistic regressions are represented in Table 3 and by distinct lines and shaded confidence intervals in Figure 2. During the total procedure the MAP < 60, MAP < 55, and < MAP50 threshold groups were all significant for the primary outcome (MAP < 60: OR = 1.002 [1.001, 1.004], *p* < 0.001; MAP < 55: OR = 1.004 [1.002, 1.006], *p* < 0.001; MAP < 50: OR = 1.006 [1.002, 1.010], *p* = 0.002. With the > 20 min group showing the highest OR in the MAP < 60 and MAP < 55 (OR = 1.473 [0.97, 2.26], *p* = 0.072; OR = 2.576 [1.464, 4.662], *p* < 0.001, respectively), while all of the time durations in the MAP < 50 were associated with AKI (1–5 min: OR = 1.421 [1.027, 1.974], *p* = 0.035; 6–10 min: OR = 1.610 [1.074, 2.42], *p* = 0.021; 11–20 min: OR = 2.356 [1.53, 3.653], *p* < 0.001; > 20 min: OR = 1.901 [1.214, 2.988], *p* = 0.005). During the anheptic phase, the overall threshold groups MAP < 60 and MAP < 55 were associated with AKI (OR = 1.011 [1.0, 1.022], *p* = 0.047 and OR = 1.023 [1.002, 1.047], *p* = 0.040. In the MAP < 50 threshold group the 11–20 min duration reached significance (OR = 5.829 [1.525, 38.07], *p* = 0.023). During the neohepatic phase the MAP < 60 threshold group reached significance (OR = 1.032 [1.01, 1.056], *p* = 0.006) with > 20 min the significant contributor (OR = 2.372 [1.073, 5.616], *p* = 0.038). No other time durations reached significance. These results are visually depicted in Figure 2, showing the association between the occurrence of AKI and the intraoperative MAP thresholds over a longer period of time during the total procedure (Fig. 2A) and the anhepatic phase (Fig. 2B). However, this association was not clearly seen in the neohepatic phase (Fig. 2C). Using risk adjusted multivariable regression analysis (Table 3) during the total duration of the procedure the MAP < 60 and MAP < 55 threshold groups remained associated with the primary outcome (OR = 1.005 [1.002, 1.008], *p* < 0.001 and OR = 1.008 [1.003‒1.013], *p* = 0.004) especially in the > 20 min group (MAP < 60: OR = 1.971 [1.16, 3.421], *p* = 0.014 and MAP < 55: OR = 3.442 [1.637, 7.571], *p* = 0.001). The MAP < 50 threshold group did not reach overall significance, but the association was seen in the 1–5 min; 6–10 min; 11–20 min duration groups (OR = 1.477 [1.007, 2.177], *p* = 0.047; OR = 1.805 [1.12, 2.923], *p* = 0.016; OR = 2.020 [1.202, 3.417], *p* = 0.008 respectively). During the anhepatic and the neohepatic phase this association did no longer reach significance.

**Table 3:** MAP thresholds and AKI.

Total Procedure MAP thresholds and the likelihood of the primary outcome AKI					
MAP threshold (mmHg)	Duration (min)	Unadjusted Odds Ratio (95% CI)	p-value	Adjusted Odds Ratio (95% CI)	*p*-value
< 60	**All**	**1.002 (1.001, 1.004)**	**<0.001**	**1.005 (1.002, 1.008)**	**<0.001**
	1‒5	a		a	
	6‒10	1.036 (0.578,1.854	0.906	1.572 (0.773, 3.226)	0.214
	11‒20	1.283 (0.771, 2.148)	0.339	1.684 (0.896, 3.21)	0.108
	> 20	1.473 (0.97, 2.26)	0.072	**1.971 (1.16, 3.421)**	**0.014**
< 55	**All**	**1.004 (1.002, 1.006)**	**<0.001**	**1.008 (1.003‒1.013)**	**0.004**
	1‒5	1.434 (0.806, 2.619)	0.228	1.749 (0.832, 3.843)	0.149
	6‒10	1.368 (0.75, 2.555)	0.314	1.971 (0.911, 4.444)	0.091
	11‒20	1.651 (0.917, 3.05)	0.100	1.713 (0.802, 3.811)	0.173
	**> 20**	**2.576 (1.464, 4.662)**	**0.001**	**3.442 (1.637, 7.571)**	**0.001**
< 50	**All**	**1.006 (1.002, 1.010)**	**0.002**	1.002 (0.994, 1.012	0.591
	**1‒5**	**1.421 (1.027, 1.974)**	**0.035**	**1.477 (1.007, 2.177)**	**0.047**
	**6‒10**	**1.610 (1.074, 2.42)**	**0.021**	**1.805 (1.12, 2.923)**	**0.016**
	**11‒20**	**2.356 (1.53, 3.653)**	**< 0.001**	**2.020 (1.202, 3.417)**	**0.008**
	**> 20**	**1.901 (1.214, 2.988)**	**0.005**	1.585 (0.908, 2.778)	0.106
**Anhepatic Phase MAP thresholds and the likelihood of the primary outcome AKI**					
< 60	**All**	**1.011 (1.0, 1.022)**	**0.047**	1.009 (0.997‒1.023)	0.165
	1‒5	1.279 (0.987, 1.66)	0.063	1.250 (0.916, 1.709)	0.160
	6‒10	0.739 (0.448, 1.201	0.228	0.552 (0.301, 0.989)	0.05
	11‒20	1.288 (0.767, 2.165)	0.337	1.519 (0.802, 2.915)	0.202
	> 20	1.401 (0.806, 1.364)	0.324	1.401 (0.796, 2.486)	0.245
< 55	**All**	**1.023 (1.002, 1.047)**	**0.040**	1.028 (1, 1.06)	0.07
	1‒5	1.049 (0.806, 1.364)	0.722	0.939 (0.685‒1.285)	0.694
	6‒10	1.392 (0.784, 2.493)	0.260	1.743 (0.85‒3.686)	0.135
	11‒20	1.408 (0.713, 2.816)	0.324	1.316 (0.548, 3.258)	0.542
	**> 20**	**2.520 (1.106, 6.246)**	**0.034**	2.763 (0.953, 9.265)	0.074
< 50	All	1.024 (0.993, 1.062)	0.162	1.023 (0.983, 1.073)	0.297
	1‒5	1.119 (0.842, 1.487)	0.437	1.023 (0.721, 1.451)	0.899
	6‒10	0.986 (0.429, 2.23)	0.974	0.954 (0.325, 2.769)	0.930
	**11‒20**	**5.829 (1.525, 38.07)**	**0.023**	**9.839 (1.794, 183)**	**0.032**
	> 20	1.166 (0.322, 4.22)	0.809	0.866 (0.161, 5.174)	0.866
**Neohepatic Phase MAP thresholds and the likelihood of the primary outcome AKI**					
< 60	**All**	**1.032 (1.01, 1.056)**	**0.006**	1.024 (0.997, 1.053)	0.085
	1‒5	0.968 (0.748, 1.253)	0.804	1.181 (0.863, 1.615)	0.298
	6‒10	1.314 (0.854,2.028)	0.215	1.383 (0.822, 2.342)	0.223
	11‒20	1.553 (0.935, 2.606)	0.091	1.304 (0.721, 2.384)	0.383
	**> 20**	**2.372 (1.073, 5.616)**	**0.038**	2.225 (0.845, 6.559)	0.120
< 55	All	1.036 (1, 1.075)	0.051	1.018 (0.976, 1.063)	0.419
	1‒5	1.167 (0.892, 1.526)	0.261	**1.418 (1.013, 1.99)**	**0.042**
	6‒10	1.092 (0.613, 1.938)	0.764	1.037 (0.513, 2.112)	0.918
	11‒20	1.183 (0.501, 2.793)	0.698	0.614 (0.228, 1.631)	0.325
	> 20	2.759 (0.761, 12.875)	0.143	2.605 (0.526, 18)	0.269
< 50	All	1.019 (0.97‒1.072)	0.456	0.994 (0.938, 1.055)	0.849
	1‒5	1.038 (0.769, 1.399)	0.809	1.098 (0.757, 1.595)	0.622
	6‒10	0.821 (0.315, 2.048)	0.675	0.864 (0.277, 2.687)	0.797
	11‒20	1.129 (0.312, 4.085)	0.849	0.664 (0.148, 2.97)	0.580
	> 20	3.387 (0.432, 68.621)	0.291	1.872 (0.161, 42)	0.624

The results for MAP < 60 mmHg, MAP < 55 mmHg, and MAP < 50 mmHg in the total patient group, the anhepatic phase, and neohepatic phase, are shown with the unadjusted and adjusted Odds Ratios (95% CI) and the p-values, with p < 0.05 considered significant.

OR, Odd Ratio; CI, Confidence Interval; MAP, Mean Arterial Pressure; BMI, Body Mass Index; NDD, Neurological Death Donation; DCD, Donation after Cardiac Death; CIT, Cold Ischemic Time; RBC, Red Blood Cells.

a The 0–5 min group was incorporated in the reference group to improve robustness of the overall model.

## Discussion

Acute kidney injury continues to be a common complication in LT, with an incidence of 47.2%, ranging from stage one in 30% of patients, Stage 2 in 10% and Stage 3 in 6%. Hypotension during liver transplantation is associated with AKI and the most critical time of this hypotension occurs during the anhepatic phase where MAP of < 60 mmHg is associated with AKI. The importance of MAP resulting in postoperative negative outcomes including AKI, has been stated numerous times.^16^^,^^17^ This current study adds to this list the importance of hypotension during the anhepatic phase which has not clearly been described before, but many clinicians suspected blood pressure management during this phase would be critical. In fact, part of the purported benefit of partial IVC cross clamp was thought to be due to the improved hemodynamics.^18^ Other indications that the anhepatic phase might be essential is the association between a decreased SvO_2_ during anhepatic phase and postoperative AKI in a retrospective study by Won Ho Kim.^19^ They used SvO_2_ as a hemodynamic parameter during LT and the association with AKI was not seen in the neohepatic phase. Also, the volume of urine during the anhepatic phase was found to be an independent predictor for postoperative AKI in a novel online AKI prediction calculator.^20^

We speculate that during the anhepatic phase a potential “triple hit” occurs on the perfusion of the kidneys. First, the cardiac output and arterial perfusion pressures of the kidney are drastically reduced due to clamping of the vena cava, decreasing the perfusion of the organ. Second, the vasopressors demand in this phase of the procedure is high, further limiting optimal organ perfusion. Finally, the caval cross-clamp increases caval pressures and venous congestion of the kidneys, only to amplify the impairment of kidney function. In a rat LT model a significant increase in the Renal Resistive Index (RRI) during the anhepatic phase was seen in a similar matter to the renal response to hemorrhagic shock^21^ and a greater RRI is also seen with an increased CVP in heart failure patients,^22^ both supportive of the hemodynamic changes during the anhepatic phase of LT. This hypothesis will need further investigation.

The results in the neohepatic phase were not anticipated, as much has been written on the post reperfusion phase, with the occurrence of post reperfusion syndrome resulting in a profound decrease in blood pressure and its association with AKI.^11^^,^^23^^,^^24^ However, those studies have not looked into the specific effects of the anhepatic phase and one could speculate that the post reperfusion syndrome is caused by the effects initiated during the anhepatic phase. This new perspective of hemodynamics during LT will need further exploration.

The surgical technique did not make a significant difference on patients’ outcome. The full cross-clamp of the vena cava was not associated with AKI, but it did reduce hypotension with a MAP < 55 mmHg altogether. Maintenance of caval flow during LT facilitates venous return to the right cardiac chamber, which reduces the risk of hemodynamic instability and preserves perfusion of the kidneys. The lack of an association between the partial caval cross-clamp and AKI might be explained because we have one surgeon who routinely performs the partial clamp, in most other cases, the full caval cross-clamp has preference. However, if the patient does not tolerate cross- clamp, thereby necessitating a partial clamp, this change of plan is indicative of the horrendous hemodynamic state at that time. On the other hand, there are other studies reporting that AKI occurred equally often in the partial clamped and the classic cross-clamped group, with no difference in five-year outcomes,^25^ and even though hemodynamic parameters were improved, the partial clamp did not protect the patient from AKI.^26^

The association between MAP thresholds and AKI was strongest in the unadjusted analysis. This association was much weaker in the adjusted analysis. This suggests that a patients’ MAP may represent more than just blood pressure and might reflect patient wellness, or it reflects the influence of unmeasured variables. Finally, hypotension might have a different MAP in the individual patient. In other words, a MAP < 60 leads to AKI in one patient, while in other patients MAP < 55 is tolerated even for longer periods of time.

The study has a number of limitations. First and foremost, it is a retrospective database over a long period and some of the relevant data was lost or not collected contemporaneously leading to imprecision. In addition, external validity may be difficult to establish as in our institution there is a preference for the full caval cross-clamp, which might differ from preferences in other centers. Thirdly, the number of patients in the lower MAP threshold groups during the anhepatic and especially the neohepatic phase were small. This became more apparent in the longer time duration subgroups (e.g., the 11–20 min and > 20 min groups), which led to a wide 95% Confidence Interval range. Future studies should include an even larger number of patients when the aim is to evaluate these subgroups to provide more statistical power. Finally, long term outcome data was not available, and AKI was a transient event in the majority of cases.^9^ While AKI is a good maker for an acute event or renal insult, we are not able to comment on longer term outcomes. Acute kidney injury can be seen as a multifactorial process,^27^ and MAP during liver transplantation is only one of the factors of importance. We have found an association between blood pressure in the anhepatic phase and AKI, but in order to demonstrate causation a prospective randomized controlled trial will be needed. To prospectively, determine if avoiding hypotension as defined as an MAP < 60 mmHg would be a large undertaking, but it would be able to address the issues of longer-term outcomes. In such a trial, how the blood pressure is managed will be critical and whether to use fluids or vasoactive agents should determine a cardiac output monitor capable of providing accurate measurements in liver transplantation surgery. It is important to realize hypotension may be a marker for severity of illness and avoiding hypotension by any definition may not impact patient outcome.

Overall, the strengths of this study are the large patient population, with accurate intraoperative hemodynamic data which gives a unique insight in the importance of intraoperative phases of LT, MAP and the association with AKI.

## Conclusion

Intraoperative hypotension is independently associated with AKI following LT. This association is seen during the anhepatic phase. Therefore, maintaining MAP above 60 mmHg during the anhepatic phase may improve kidney function after liver transplantation.

## Declaration of competing interest

The authors declare no conflicts of interest.

## Appendix Supplementary materials

Image, application 1
