# Supplementary material for: Intraoperative hypotension during critical phases of liver transplantation and its impact on acute kidney injury: a retrospective cohort study
Source: Braz J Anesthesiol. 2024 Oct 16;74(6):844566. doi: 10.1016/j.bjane.2024.844566 (PMC11541844; doi:10.1016/j.bjane.2024.844566)
Supplement: Supplementary file 1 [file mmc1.pdf]

University Health Network
Research Ethics Board
700 University Ave, 4th Floor
Toronto, Ontario, M5G 1Z5
Phone: (416) 581-7849

NOTIFICATION OF REB AMENDMENT APPROVAL

Date: September 5, 2023

To: Stuart A McCluskey
Toronto General Hospital, Eaton Building, 200
Elizabeth St., 3rd Floor, North Wing, Room 3EN-405,
Toronto, Ontario, Canada, M5G 2C4

Re: 20-5974
Acute Kidney Injury following Liver Transplantation: A
Retrospective Study

REB Review Type: Delegated
REB Initial Approval Date: December 4, 2020
REB Amendment Approval Date: September 5, 2023
REB Expiry Date: December 4, 2023

Documents Approved:

Document Name	Version Date	Version ID
Protocol	August 29, 2023	Version 3

The University Health Network Research Ethics Board has reviewed and approved the Amendment (20-5974.3) for the above mentioned study.

The Amendment to extend data collection from October 31st, 2020 to June 1st, 2022 and increase sample size from approximately 1900 to 2900 has been approved.

Please note that no further extensions in the study period or increases in sample size will be granted in the future.

Best wishes on the successful completion of your project.

Sincerely,
Wesley Ghent
Ethics Coordinator, University Health Network Research Ethics Board
Approved and Digitally signed by Wesley Ghent on September 5, 2023 at 11:24 AM

For: Morris Sherman
Co-Chair, University Health Network Research Ethics Board

The UHN Research Ethics Board operates in compliance with the Tri-Council Policy Statement; ICH Guideline for Good Clinical Practice E6; Ontario Personal Health Information Protection Act (2004); Part C Division 5 of the Food and Drug Regulations; Part 4 of the Natural Health Products Regulations and the Medical Devices Regulations of Health Canada.
